# Role of Etiology in Hepatocellular Carcinoma Patients Treated with Lenvatinib: A Counterfactual Event-Based Mediation Analysis

**DOI:** 10.3390/cancers15020381

**Published:** 2023-01-06

**Authors:** Rodolfo Sacco, Daryl Ramai, Raffaella Tortora, Giovan Giuseppe di Costanzo, Michela Emma Burlone, Mario Pirisi, Piera Federico, Bruno Daniele, Marianna Silletta, Paolo Gallo, Caterina Cocuzza, Maurizio Russello, Giuseppe Cabibbo, Gabriele Rancatore, Silvia Cesario, Gianluca Masi, Luca Marzi, Andrea Mega, Alessandro Granito, Giulia Pieri, Edoardo G. Giannini, Rosa Paolillo, Gennaro Gadaleta-Caldarola, Vincenzo Dadduzio, Guido Giordano, Luca Giacomelli, Simonetta Papa, Matteo Renzulli, Marcello Maida, Michele Ghidini, Mauro Borzio, Antonio Facciorusso

**Affiliations:** 1 Gastroenterology and Endoscopy Unit, Department of Surgical and Medical Sciences, University of Foggia, 71100 Foggia, Italy; 2 Gastroenterology and Hepatology, University of Utah, Salt Lake City, UT 84112, USA; 3 Liver Unit, Department of Transplantation, Cardarelli Hospital, 80100 Naples, Italy; 4 Department of Internal Medicine, AOU “Maggiore Della Carità”, 28100 Novara, Italy; 5 Medical Oncology Unit, Ospedale del Mare, 80100 Naples, Italy; 6 Division of Medical Oncology, Policlinico Universitario Campus Bio-Medico, 00128 Rome, Italy; 7 Clinical Medicine and Hepatology Unit, Campus Bio-Medico University, 00128 Rome, Italy; 8 Liver Unit, ARNAS Garibaldi-Nesima, 95100 Catania, Italy; 9 Section of Gastroenterology & Hepatology, Department of Health Promotion, Mother and Child Care, Internal Medicine and Medical Specialties, PROMISE, University of Palermo, 90121 Palermo, Italy; 10 Unit of Medical Oncology, AOU Pisana, Santa Chiara Hospital, 56121 Pisa, Italy; 11 Gastroenterology Unit, Bolzano Regional Hospital, 39100 Bolzano, Italy; 12 Division of Internal Medicine, Hepatobiliary and Immunoallergic Diseases, S. Orsola-Malpighi Hospital, IRCCS AOU di Bologna, 40121 Bologna, Italy; 13 Gastroenterology Unit, Department of Internal Medicine, University of Genova, Istituto di Ricovero e Cura a Carattere Scientifico (IRCCS) Ospedale Policlinico San Martino, 16100 Genova, Italy; 14 Medical Oncology Unit, Mons. A.R. Dimiccoli Hospital, 76121 Barletta, Italy; 15 Medical Oncology and Biomolecular Therapy Unit, Department of Surgical and Medical Sciences, University of Foggia, 71100 Foggia, Italy; 16 Polistudium SRL, 20135 Milan, Italy; 17 Department of Radiology, IRCCS AOU di Bologna, 40121 Bologna, Italy; 18 Gastroenterology and Endoscopy Unit, S. Elia-Raimondi Hospital, 93100 Caltanissetta, Italy; 19 Division of Medical Oncology, Fondazione IRCCS Ca’ Granda, Ospedale Maggiore Policlinico, 20100 Milano, Italy; 20 Gastroenterologia ed Endoscopia Digestiva, Centro Diagnostico Italiano, 20100 Milan, Italy

**Keywords:** HCC, survival, progression, liver cancer, mediation analysis, viral etiology, HCV, HBV, nonviral liver disease

## Abstract

**Simple Summary:**

Hepatocellular carcinoma is the fifth most common cancer worldwide. For patients with advanced hepatocellular carcinoma, therapeutic options are limited. Lenvatinib has proven to be an effective option for treating advanced diseases. For patients treated with lenvatinib, our study showed that the etiology of liver disease plays a significant role in overall survival. Patients whose liver disease was driven by nonviral etiologies showed longer survival than viral etiologies. These results are important for clinical decision-making between patients and clinicians.

**Abstract:**

Background: Whether the etiology of underlying liver disease represents a prognostic factor in patients with hepatocellular carcinoma (HCC) treated with lenvatinib is still a matter of debate. This study investigates whether the viral etiology of HCC plays a prognostic role in overall survival (OS). Methods: Data derived from a multicenter series of 313 HCC patients treated with lenvatinib between 2019 and 2022 were analyzed. Actuarial survival estimates were computed using the Kaplan–Meier method and compared with the log-rank test. We performed an event-based counterfactual mediation analysis to estimate direct (chronic inflammation and immunosuppression), indirect (tobacco smoking, alcohol use, illicit drug abuse with injections), and the total effect of viral etiology on OS. Results were expressed as hazard ratio (HR) and 95% CI. Results: Median OS was 21 months (95% CI: 20–23) in the group with other etiologies and 15 months (14–16) in the group with viral etiology (*p* < 0.0001). The total effect of viral etiology was associated with OS (HR 2.76, 1.32–5.21), and it was mainly explained by the pure direct effect of viral etiology (HR 2.74, 1.15–4.45). By contrast, its total indirect effect was not associated with poorer survival (HR 1.05, 0.82–2.13). These results were confirmed when considering tobacco, alcohol consumption, or injection drug abuse as potential mediators. Median progression-free survival was 9 months (8–10) in patients with other etiologies and 6 months (5–7) in patients with viral etiology (*p* < 0.0001). No difference in terms of adverse event rate was observed between the two groups. Conclusions: Patients affected by HCC with nonviral etiology treated with lenvatinib exhibit longer survival than those with viral etiology. This finding may have relevance in the treatment decision-making process.

## 1. Introduction

Hepatocellular carcinoma (HCC) is the fifth most common type of cancer and the leading cause of mortality in patients with cirrhosis around the world [1].

In developed countries, the proportion of patients diagnosed with early HCC suitable for curative treatments is growing. However, a high number of cases are detected in the presence of portal vein thrombosis and/or extrahepatic spread, which are common indications for systemic therapies [2].

For subjects with unresectable advanced HCC who would not benefit from surgical and locoregional treatments, the oral multikinase inhibitor sorafenib (Nexavar^®^, Bayer, Leverkusen, Germany) was the first and only systemic treatment for more than a decade [2,3,4]. However, the narrow therapeutic window and the high rate of progression constitute major pitfalls of sorafenib therapy. More recently, other tyrosine kinase inhibitors (TKIs) have been approved as first-line (lenvatinib) or second-line (regorafenib and cabozantinib) treatments. Additional treatments have been approved, including combination immunotherapy (Atezolizumab) and monoclonal antibody (bevacizumab) as first-line therapy or monoclonal antibody ramucirumab as second-line therapy for patients with alpha-fetoprotein (AFP) ≥ 400 ng/mL [5,6,7]. Given the poor prognosis and evolving therapeutic landscape, research on treatment selection for HCC is a major unmet need [5,8].

Lenvatinib (Lenvima^®^, Eisai GmbH, Germany) is a multi-TKI endowed with one of the most potent anti-tumoral activities among other TKIs [9]. A phase III multicenter randomized-controlled trial (RCT) demonstrated the non-inferiority of lenvatinib when compared with sorafenib in terms of overall survival (OS) and a similar safety profile in untreated advanced HCC patients [10]. Since then, several real-life series and a recent meta-analysis have confirmed a similar OS and more favorable progression-free survival (PFS) and objective response rate (ORR) with lenvatinib compared with sorafenib as first-line therapy [11,12,13,14,15,16,17,18,19,20].

Due to the complex pathophysiology involved in HCC development, several efforts have been made to elucidate the role of HCC etiology and its effect on response rate and survival outcomes. To this end, nonalcoholic steatohepatitis (NASH) etiology has been associated with a worse prognosis in patients treated with immune checkpoint inhibitors, likely due to a different hepatic microenvironment that consequently affects treatment response [21,22].

On the other hand, a recent multicenter series showed that both OS and PFS were prolonged in patients with NASH treated with lenvatinib [12].

To better define the prognostic role of liver disease etiology, we performed a counterfactual event-based mediation analysis through a large Italian multicenter series of patients diagnosed with HCC and treated with first-line therapy lenvatinib. We determined the effect of direct (reflecting chronic inflammation and immunosuppression), indirect (reflecting increased alcohol and tobacco consumption and illicit drug abuse), and total liver disease etiology on patient survival.

## 2. Patients and Methods

### 2.1. Patients

From a retrospective analysis of a prospectively collected Italian multicenter database, data were retrieved from HCC patients treated between May 2019 and Jan 2022 with lenvatinib as first-line therapy for stage B or C at the Barcelona Clinic Liver Centre (BCLC). Patients treated with lenvatinib were not deemed eligible for surgical or locoregional therapies. HCC was diagnosed histologically and/or confirmed using international clinical guidelines [2,23]. Patients did not receive previous systemic therapy.

Inclusion criteria for enrollment in this study included Eastern Cooperative Oncology Group (ECOG) performance score ≤ 2; Child-Pugh class ≤ B7; platelet levels ≥ 50 × 10^9^/L; hemoglobin ≥ 8.5 g/dL and prothrombin time/international normalized ratio ≤ 2; adequate renal function (serum creatinine ≤ 1.5 times the upper limit of the normal range). Sporadic and minor deviations of patients’ inclusion criteria were permitted based on clinical judgment and clinical practice of each center. All patients provided written informed consent before their enrollment in this study. The ethics committee of the leading center (Ospedali Riuniti Azienda Ospedaliero-Universitaria, Foggia, Italy; protocol number: 137/CE/2021 of 19-10-2021) approved this study. HCC was considered to have viral etiology if the patient tested positive for hepatitis C virus (HCV) or hepatitis B virus (HBV), according to the practice of the treating center. All HCC patients with negative viral tests were considered to have a nonviral etiology.

### 2.2. Treatment

Oral lenvatinib was administered once daily as described in the REFLECT trial [9]; patients received 12 mg if baseline body weight was ≥60 kg or 8 mg if baseline body weight was <60 kg.

Tumor response was assessed through computed tomography (CT) scan/magnetic resonance imaging (MRI) every eight weeks or as clinically indicated. Tumor response was assessed according to the modified RECIST criteria [24].

Treatment with lenvatinib was interrupted in the presence of tumor progression or unacceptable toxicity. Treatment interruptions and dose reductions were considered in managing adverse events (AEs).

### 2.3. Outcomes

The primary outcome was OS, computed from the start of treatment until death of any cause or censoring. Secondary outcomes were PFS (defined as the time elapsed from treatment to radiological evidence of progression), tumor response expressed in terms of objective response (defined as complete response + partial response), mean treatment duration and AE rate. The severity of AEs was graded following the common terminology criteria for adverse events (CTCAE) v4.013.

### 2.4. Statistical Analysis

Categorical variables were described as frequencies and percentages, while continuous variables as the median and interquartile range (IQR). Time-to-event data were estimated from the start of treatment with lenvatinib by Kaplan–Meier plots and compared using log-rank tests.

The inferential analysis for time-to-event data was conducted using Cox univariate and multivariate regression models to estimate HRs and 95% CI. The multivariate models consistently included statistically significant variables from the univariate analysis.

**Figure 1:**
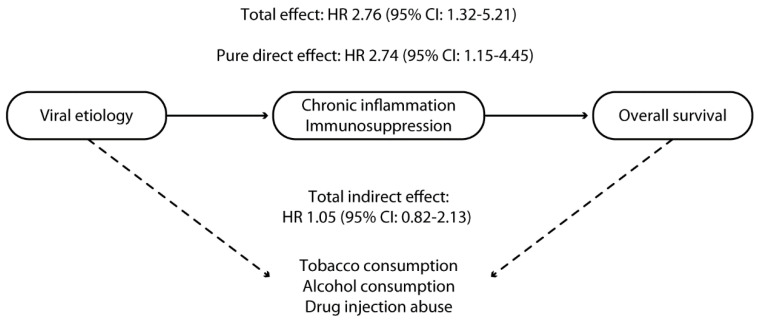
Directed acyclic graph reporting the assumed causal relationship between viral etiology and survival in patients with hepatocellular carcinoma treated with lenvatinib. The continuous line represents the direct effect of chronic inflammation and immunosuppression; the dotted line represents the indirect effect mediated by tobacco, alcohol consumption, and injection drug abuse.

Even though HCV or HBV viral infection does not induce alcohol or tobacco consumption or illicit drug abuse, the higher rates of these abuses in HCV or HBV-infected patients [25] could reflect higher rates of risky behavior in this population. This suggests the need to consider these consumptions as confounding factors and potential mediators of the impact of viral etiology on survival outcomes, as depicted in the directed acyclic graph (Figure 1).

The pure direct effect (PDE), total indirect effect (TIE), and total effect (TE) of viral etiology on survival outcomes were estimated using mediation analysis [26]. In this analysis, exposure was HBV or HCV infection, and mediators were alcohol or tobacco consumption and drug abuse. TIE reflected the effect of the above-reported mediators on the primary outcome. In contrast, PDE reflected the effect of chronic inflammation and immunosuppression directly related to viral replication in the absence of other unmeasured mediators [26]. Therefore, the PDE of viral infection reflects how much the HR would change if all participants presented with viral etiology vs. if none of the participants presented with viral etiology. Nevertheless, the values of mediators were kept at their reported levels.

On the other hand, the TIE reflects how much the HR would change if the exposure was kept at the observed level; however, mediator values were changed from the level they would have if participants had a viral etiology compared with the value they would have if none of the participants had a viral etiology. Finally, TE reflects how much the HR would change if all participants had a viral etiology vs. none of them; mediator values were also changed from the values they would have if no participants had a viral etiology to the values they would have if all participants did. The TE was calculated by multiplying PDE and TIE.

The analysis was performed using R Statistical Software (Foundation for Statistical Computing, Vienna, Austria), and significance was established at the 0.05 level (two-sided).

## 3. Results

### 3.1. Baseline Characteristics of the Patients

The clinical and demographic characteristics of enrolled patients are summarized in Table 1. Out of 313 patients treated with lenvatinib as first-line systemic therapy, 98 (31.3%) did not have a viral etiology underlying their liver disease, whereas 215 (68.7%) were HBV+ (ve) or HCV+ (ve). Baseline characteristics were well balanced between the two groups.

The median age was 74 years (IQR 56–82) in patients with other etiologies and 72 (IQR 58–84) years in patients with viral etiology (*p* = 0.39). Most patients were male in both groups (*p* = 0.77), and 80.6% and 80.9% of subjects presented with ECOG PS 0 in the two groups, respectively (*p* = 0.94). The vast majority of patients were in Child-Pugh stage A in both groups (87.7% versus 84.6%, *p* = 0.46). Fifty-nine patients (60.2%) with other etiologies and 133 patients (61.9%) with viral etiology presented with BCLC stage C HCC (*p* = 0.39). Median AFP was 255.9 IU/mL (IQR 142.1–570) and 250.5 IU/mL (139.2–615) in the two groups, respectively (*p* = 0.71). More than half of patients (59.1% and 63.2% in the two groups, respectively; *p* = 0.67) had previously received radical or locoregional therapies.

Among the patients with viral etiology-related liver disease, 184 (85.5%) received antiviral treatment with a sustained virological response. Among patients in the nonviral group, 57 had an alcohol etiology, 30 had a NASH etiology, and 11 with other etiologies.

### 3.2. Overall Survival

**Figure 2:**
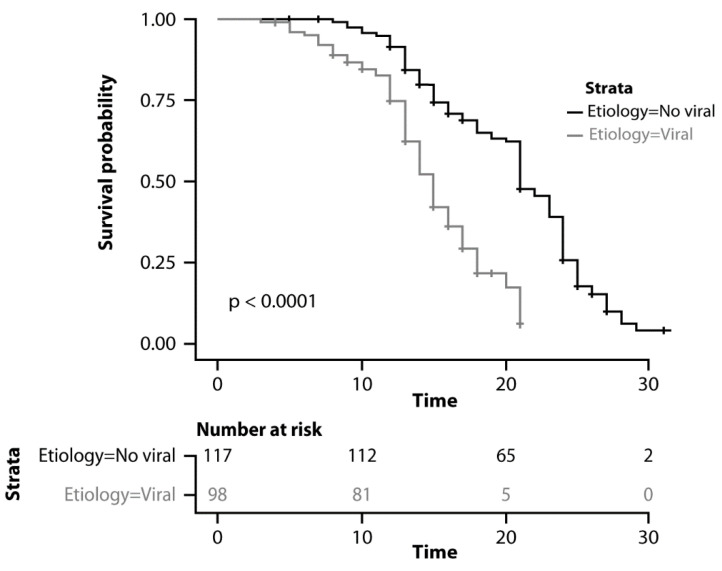
Kaplan–Meyer curves comparing overall survival.

Overall, the median OS was 18 months (95% CI: 17–20). Median OS was 21 months (95% CI: 20–23) in the group with other etiologies and 15 months (95% CI: 14–16) in the group with viral etiology (*p* < 0.0001; Figure 2).

As reported in Table 2, viral etiology of the underlying liver disease (*p* < 0.0001), Child-Pugh score (*p* < 0.0001), AFP > 400 UI/mL (*p* = 0.01), BCLC stage (*p* = 0.001), and ECOG PS (*p* = 0.02) resulted as significant predictors of OS in univariate analysis. Viral etiology (HR 2.76, 95% CI 1.32–5.21; *p* = 0.002), Child-Pugh stage (HR 2.64, 95% CI 1.61–4.44; *p* < 0.001), AFP > 400 UI/mL (HR 1.71, 95% CI 1.15–3.2; *p* = 0.01), and BCLC stage (HR 2.15, 95% CI 1.35–3.88; *p* = 0.02) were confirmed as significant predictors in multivariate analysis (Table 2).

No difference was observed in the viral etiology group between patients with HBV or HCV-related liver disease (*p* = 0.32). No difference was observed within the viral etiology group between patients with sustained virological response versus those who were not treated with antiviral therapy (HR 0.78, 95% CI 0.61–1.34; *p* = 0.14). Additionally, no difference was observed between patients with alcohol-related and NASH-related HCC (HR 1.21, 95% CI 0.78–1.54; *p* = 0.43).

The TE of viral etiology was significantly associated with an almost three-fold increased mortality risk (HR 2.76, 95% CI 1.32–5.21). As reported in Figure 1, this increased risk was mainly explained by the PDE of viral etiology (HR 2.74, 95% CI: 1.15–4.45). By contrast, the TIE of viral etiology (signifying the effect of alcohol consumption, tobacco consumption, and injection drug abuse) did not show a statistically significant association with worse survival outcomes (HR 1.05, 95% CI: 0.82–2.13) (Figure 1).

As reported in Table 3, when tobacco consumption was considered as a potential mediator of the association between viral etiology and OS, similar results were found with PDE, TIE, and TE of 1.86 (95% CI 1.16–2.45), 1.02 (95% CI 0.78–1.34), and 2.62 (95% CI 1.15–3.45), respectively. Similar results were found when alcohol consumption was considered as the only potential mediator, with PDE, TIE, and TE estimates of 1.67 (95% CI 1.13–2.5), 1.05 (95% CI 0.82–2.13), and 2.61 (95% CI 1.15–3.45), respectively (Table 3). When drug injection abuse was considered as the only potential mediator, PDE, TIE, and TE were 1.59 (95% CI 1.14–2.34), 1.13 (95% CI 0.88–2.43), and 2.60 (95% CI 1.15–3.34). 

### 3.3. Progression-Free Survival

**Figure 3:**
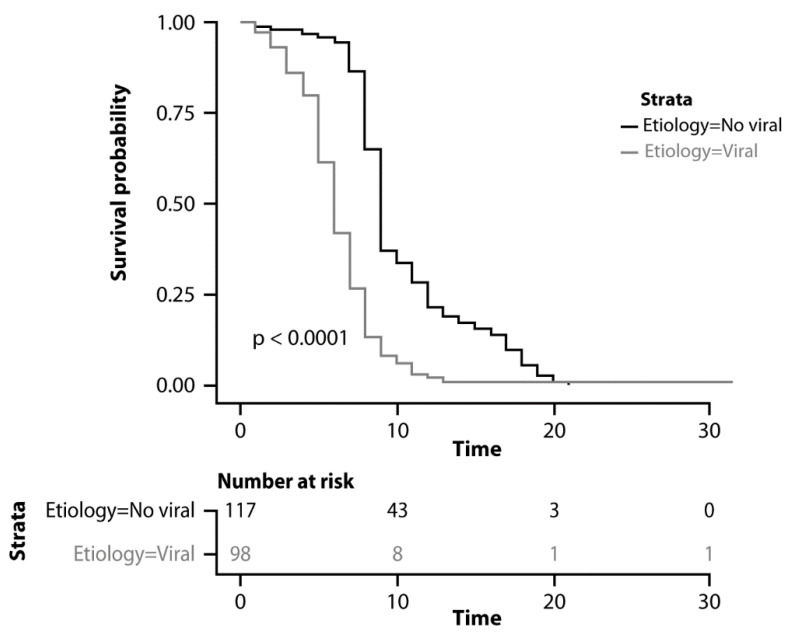
Kaplan–Meyer curves comparing progression-free survival.

Overall, the median PFS was 7 months (95% CI: 6-9). As reported in Figure 3, the median PFS was 9 months (95% CI: 8–10) in patients with other etiologies and 6 months (95% CI: 5–7) in patients with viral etiology (*p* < 0.0001).

Following univariate analysis, viral etiology (*p* < 0.0001), Child-Pugh score (*p* = 0.04), and AFP > 400 IU/mL (p = 0.003) were found to be significant predictors of PFS (Table 4). Viral etiology (HR 2.54, 95% CI 1.54–4.89; *p* = 0.004) and AFP > 400 IU/mL (HR 1.72, 95% CI 1.05–2.6; *p* = 0.05) were confirmed as significant prognostic factors following multivariate analysis. Again, no difference was observed between HBV+ and HCV+ patients (HR 1.01, 95% CI 0.6–1.8; *p* = 0.76) and between patients with sustained virological response versus patients who did not undergo the antiviral treatment (HR 0.65, 95% CI 0.54–1.18; *p* = 0.11). Additionally, no difference was observed between patients with alcohol-related and NASH-related HCC (HR 1.43, 95% CI 0.65–1.92; *p* = 0.49).

### 3.4. Other Secondary Outcomes

HCC etiology was associated with differences in the rate of tumor response. The objective response rate was 42.1% in patients with other etiologies and 30.3% in patients with viral disease (*p* = 0.05).

The mean treatment duration was 13 months (range: 10–18 months). AEs of any grade were reported in 292 patients (93.3%), of which 89 (91.0%) were patients of nonviral etiology, and 203 (94.4%) were patients with viral etiology (*p* = 0.23). The most frequent AEs were hand–foot skin toxicity (16.4%), hypertension (13.2%), and diarrhea (10.2%). Grade 3/4 AEs were similar between both groups (14.9% vs. 15.1%; *p* = 0.65).

## 4. Discussion

Given the unfavorable results with sorafenib therapy, other first-line treatment options such as lenvatinib, as well as immune checkpoint inhibitors, or second lines therapies such as regorafenib and cabozantinib, have been introduced in clinical practice to treat unresectable advanced HCC [7,11,27,28]. In particular, lenvatinib showed promising results in the pivotal multicenter RCT and in several real-life clinical series [10,11,12,13,14,15,16,17,18,19,20,29,30]. However, evidence is still limited on the prognostic factors that might impact patient survival. A recent multicenter retrospective series found longer OS and PFS in patients with NASH treated with lenvatinib compared with sorafenib [12]. In a study on 67 HCC patients treated with lenvatinib, nonviral etiology was associated with improved objective response, PFS, and OS compared with nonviral etiology [31]. However, these findings were challenged by those reported in other studies, which demonstrated no difference in the efficacy of lenvatinib between viral and nonviral etiologies [15,32]. Due to inconclusive results in the reported literature, we evaluated the role of viral versus nonviral etiology in HCC patients treated with lenvatinib.

The main result of our analysis showed that first-line lenvatinib was associated with favorable clinical outcomes and an overall manageable toxicity profile, in line with the findings of the REFLECT clinical trial and of the real-life study by Rimini et al. [10,20]. 

Furthermore, in our study, lenvatinib was associated with improved effectiveness in HCC of nonviral etiology, compared with HCC with viral etiology, without any worsening in toxicity. Overall, our results were similar to those reported in another study by Rimini et al., who analyzed a large international cohort of patients (n = 1232) treated with lenvatinib [12]. In this analysis, NASH-HCC was associated with longer mOS than viral etiology [22.2 versus 15.1 months; HR 0.69; 95% CI: 0.56–0.85; *p* = 0.0006] and with longer mPFS (7.5 versus 6.5 months; HR 0.84; 95% CI: 0.71–0.99; *p* = 0.0436). These findings provide potentially relevant evidence for decision-making among patients and healthcare providers, particularly in selecting systemic treatment for unresectable advanced HCC. Indeed, data from preclinical and clinical models suggest potentially lower responsiveness of NASH-HCC to immunotherapy, perhaps due to distinctive phenotypic characteristics of T cells [22]. The inflammatory pathways induced in the milieu of NASH could also represent a potential issue for other treatments [33,34]. Therefore, the high effectiveness of lenvatinib in HCC of nonviral etiology can be considered when selecting treatment options in this setting.

We also performed a counterfactual event-based mediation analysis to understand the potential underlying mechanisms of the improved effectiveness of lenvatinib in HCC of nonviral etiology compared with HCC with viral etiology. Counterfactual event-based mediation analysis represents an approach that reveals how much effect viral etiology has on survival outcomes directly (attributable to the viral etiology itself) and indirectly (how much is mediated by other factors, such as tobacco or alcohol consumption or drug abuse). The indirect effect of viral etiology, reflecting the effects of higher alcohol/tobacco consumption or drug abuse demonstrated in HCC patients with viral etiology [35,36], was not significantly associated with mortality; therefore, our findings are based on a PDE probably related to chronic inflammation and immunosuppression induced by replication of HBV or HCV viruses. To our knowledge, no other study has assessed the prognostic role of viral etiology in these patients and the impact of potential confounders through a robust methodology. On the other hand, previous reports have pointed out the role of alcohol or tobacco consumption on patient survival [37,38].

No difference was observed in terms of OS and PFS within the viral etiology group between patients with sustained virological response and patients not treated with antiviral therapy (*p* = 0.14). This is probably due to the limited number of patients in the latter subgroup (patients who never underwent any antiviral treatments, n = 31). Therefore, based on our study, no assumption on the prognostic impact of antiviral treatment in HCC patients can be drawn.

The worse survival observed in viral-related HCC could be due to several potential carcinogenetic mechanisms of hepatitis viruses, such as direct oncogenesis from the integration of the viral genome in the hepatocyte or induced chronic inflammation [39].

Nevertheless, our study has limitations. First, we performed a larger retrospective observational study which is subject to selection bias. Second, the multicenter nature of the study did not allow the centralization of imaging. Furthermore, treatment and re-evaluation criteria were used according to the clinical practice of each center in accordance with national guidelines. Third, a classification bias due to under-reported use of alcohol, tobacco, or drug abuse could represent a limitation, as well as the lack of subgroup analysis in HCC patients with nonviral etiology (e.g., NASH) to assess the possible effect of mediators in this group.

## 5. Conclusions

In conclusion, our results suggest that first-line lenvatinib is effective in patients with advanced HCC of either nonviral or viral etiologies. Lenvatinib showed higher treatment efficacy in cases of nonviral etiology compared to viral etiology.

Viral etiology may be considered an independent prognostic factor in HCC patients treated with lenvatinib, mainly due to its direct effect on chronic inflammation and immunosuppression. The etiology of HCC could help better tailor treatment strategies, such as patients with advanced HCC.

## Figures and Tables

**Table 1:** Patients’ baseline characteristics.

Variable	Nonviral Etiology (98 Patients)	Viral Etiology (215 Patients)	*p*-Value
Age (years)	74 (56–82)	72 (58–84)	0.39
Gender (male)	77 (78.5%)	172 (80%)	0.77
ECOG PS 0	79 (80.6%)	174 (80.9%)	0.94
Child-Pugh A	86 (87.7%)	182 (84.6%)	0.46
BCLC: • Stage B • Stage C	29 (39.8%) 59 (60.2%)	82 (38.1%) 133 (61.9%)	0.39
AFP (UI/mL)	255.9 (142.1–570)	250.5 (139.2–615)	0.71
Sustained virological response *	–	184 (85.5%)	–
Previous radical or locoregional therapy	58 (59.1%)	136 (63.2%)	0.67

Values are expressed as median (interquartile range) or absolute numbers (percentages), as appropriate. * Only in the group with viral etiology. AFP, alpha-fetoprotein; BCLC, Barcelona Cancer of the Liver Clinic; ECOG, Eastern Cooperative Oncology Group; PS, Performance Status.

**Table 2:** Cox univariate/multivariate regression for overall survival.

Variables	Univariate Analysis		Multivariate Analysis	
	Hazard Ratio (95% CI)	*p*-Value	Hazard Ratio (95% CI)	*p*-Value
Age (reference ≤ 65 years)	0.86 (0.56–1.45)	0.26		
Gender (reference Female)	1.06 (0.78–2.3)	0.54		
Etiology (reference nonviral)	2.34 (1.26–4.31)	<0.001	2.76 (1.32–5.21)	0.002
Child-Pugh (reference A)	2.39 (1.88–4.11)	<0.001	2.64 (1.61–4.44)	<0.001
AFP (reference ≤ 400 UI/mL)	2.08 (1.09–3.1)	0.01	1.71 (1.15–3.2)	0.01
BCLC (reference B)	2.2 (1.28–4.3)	0.001	2.15 (1.35–3.88)	0.02
Hepatitis virus (reference HBV) *	1.11 (0.8–2.3)	0.32		
ECOG PS (reference 0)	1.75 (1.07–2.8)	0.02	1.23 (0.65–4.81)	0.14

* Analysis restricted to patients with viral etiology. AFP, alpha-fetoprotein; BCLC, Barcelona Cancer of the Liver Clinic; CI 95%, confidence interval 95%; ECOG, Eastern Cooperative Oncology Group; HBV, hepatitis B virus; PS, Performance Status.

**Table 3:** Direct, indirect, and total effects of viral etiology on overall survival.

Effects	Hazard Ratio (95% CI)
Current tobacco consumption as the only mediator: • Pure direct effect • Total indirect effect • Total effect	1.86 (1.16–2.45) 1.02 (0.78–1.34) 2.62 (1.15–3.45)
Current alcohol consumption as the only mediator: • Pure direct effect • Total indirect effect • Total effect	1.67 (1.13–2.5) 1.05 (0.82–2.13) 2.61 (1.15–3.45)
Current injection drug abuse as the only mediator: • Pure direct effect • Total indirect effect • Total effect	1.59 (1.14–2.34) 1.13 (0.88–2.43) 2.60 (1.15–3.34)

**Table 4:** Cox univariate/multivariate regression for progression-free survival.

Variables	Univariate Analysis		Multivariate Analysis	
	Hazard Ratio (95% CI)	*p*-Value	Hazard Ratio (95% CI)	*p*-Value
Age (reference ≤ 65 years)	0.89 (0.64–1.75)	0.31		
Gender (reference Female)	1.16 (0.65–2.1)	0.58		
Etiology (reference nonviral)	2.49 (1.51–4.56)	<0.001	2.54 (1.54–4.89)	0.004
Child-Pugh (reference A)	1.54 (1.08–2.11)	0.04	1.61 (0.89–3.42)	0.11
AFP (reference ≤ 400 UI/mL)	2.18 (1.21–4.23)	0.003	1.72 (1.05–2.6)	0.05
BCLC (reference B)	1.8 (0.88–2.3)	0.09		
Hepatitis virus (reference HBV) *	1.01 (0.6–1.8)	0.76		
ECOG PS (reference 0)	1.45 (0.87–2.9)	0.42		

* Analysis restricted only to patients with viral etiology. AFP, alpha-fetoprotein; BCLC, Barcelona Cancer of the Liver Clinic; ECOG, Eastern Cooperative Oncology Group; HBV, hepatitis B virus; PS, Performance Status.

## Data Availability

The data presented in this study are available upon request from the corresponding author.

## References

[1] El-Serag H.B. (2011). Hepatocellular carcinoma. N. Engl. J. Med..

[2] Heimbach J.K., Kulik L.M., Finn R.S., Sirlin C.B., Abecassis M.M., Roberts L.R., Zhu A.X., Murad M.H., Marrero J.A. (2018). AASLD guidelines for the treatment of hepatocellular carcinoma. Hepatology.

[3] Llovet J.M., Ricci S., Mazzaferro V., Hilgard P., Gane E., Blanc J.F., de Oliveira A.C., Santoro A., Raoul J.L., Forner A. (2008). Sorafenib in advanced hepatocellular carcinoma. N. Engl. J. Med..

[4] Ponziani F.R., Bhoori S., Germini A., Bongini M., Flores M., Sposito C., Facciorusso A., Gasbarrini A., Mazzaferro V. (2016). Inducing tolerability of adverse events increases sorafenib exposure and optimizes patient’s outcome in advanced hepatocellular carcinoma. Liver. Int..

[5] Laface C., Fedele P., Maselli F.M., Ambrogio F., Foti C., Molinari P., Ammendola M., Lioce M., Ranieri G. (2022). Targeted therapy for hepatocellular carcinoma: Old and new opportunities. Cancers.

[6] Zhou M., Liu B., Shen J. (2022). Immunotherapy for hepatocellular carcinoma. Clin. Exp. Med..

[7] Cabibbo G., Aghemo A., Lai Q., Masarone M., Montagnese S., Ponziani F.R., Italian Association for the Study of the Liver (AISF) (2022). Optimizing systemic therapy for advanced hepatocellular carcinoma: The key role of liver function. Dig. Liver. Dis..

[8] Falette Puisieux M., Pellat A., Assaf A., Ginestet C., Brezault C., Dhooge M., Soyer P., Coriat R. (2022). Therapeutic management of advanced hepatocellular carcinoma: An updated review. Cancers.

[9] Decraecker M., Toulouse C., Blanc J.F. (2021). Is There still a place for tyrosine kinase inhibitors for the treatment of hepatocellular carcinoma at the time of immunotherapies? A focus on lenvatinib. Cancers.

[10] Kudo M., Finn R.S., Qin S., Han K.H., Ikeda K., Piscaglia F., Baron A., Park J.W., Han G., Jassem J. (2018). Lenvatinib versus sorafenib in first-line treatment of patients with unresectable hepatocellular carcinoma: A randomised phase 3 non-inferiority trial. Lancet.

[11] Facciorusso A., Tartaglia N., Villani R., Serviddio G., Ramai D., Mohan B.P., Chandan S., Abd El Aziz M.A., Evangelista J., Cotsoglou C. (2021). Lenvatinib versus sorafenib as first-line therapy of advanced hepatocellular carcinoma: A systematic review and meta-analysis. Am. J. Trans. Res..

[12] Rimini M., Kudo M., Tada T., Shigeo S., Kang W., Suda G., Jefremow A., Burgio V., Iavarone M., Tortora R. (2021). Nonalcoholic steatohepatitis in hepatocarcinoma: New insights about its prognostic role in patients treated with lenvatinib. ESMO Open.

[13] Burgio V., Iavarone M., Di Costanzo G.G., Marra F., Lonardi S., Tamburini E., Piscaglia F., Masi G., Celsa C., Foschi F.G. (2021). Real-life clinical data of lenvatinib versus sorafenib for unresectable hepatocellular carcinoma in Italy. Cancer Manag. Res..

[14] Kuo Y.H., Lu S.N., Chen Y.Y., Kee K.M., Yen Y.H., Hung C.H., Hu T.H., Chen C.H., Wang J.H. (2021). Real-world Lenvatinib versus sorafenib in patients with advanced hepatocellular carcinoma: A propensity score matching analysis. Front. Oncol..

[15] Hiraoka A., Kumada T., Tada T., Tani J., Kariyama K., Fukunishi S., Atsukawa M., Hirooka M., Tsuji K., Ishikawa T. (2021). Real-life Practice Experts for HCC (RELPEC) Study Group and HCC 48 Group (hepatocellular carcinoma experts from 48 clinics in Japan). Efficacy of lenvatinib for unresectable hepatocellular carcinoma based on background liver disease etiology: Multi-center retrospective study. Sci. Rep..

[16] Tada T., Kumada T., Hiraoka A., Atsukawa M., Hirooka M., Tsuji K., Ishikawa T., Takaguchi K., Kariyama K., Itobayashi E. (2021). Real-life Practice Experts for HCC (RELPEC) Study Group and the HCC 48 Group (hepatocellular carcinoma experts from 48 clinics in Japan). Impact of modified albumin-bilirubin grade on survival in patients with HCC who received lenvatinib. Sci. Rep..

[17] Hiraoka A., Kumada T., Kariyama K., Takaguchi K., Atsukawa M., Itobayashi E., Tsuji K., Tajiri K., Hirooka M., Shimada N. (2019). Real-life Practice Experts for HCC (RELPEC) Study Group, HCC 48 Group (hepatocellular carcinoma experts from 48 clinics in Japan). Clinical features of lenvatinib for unresectable hepatocellular carcinoma in real-world conditions: Multicenter analysis. Cancer Med..

[18] Hiraoka A., Kumada T., Kariyama K., Takaguchi K., Itobayashi E., Shimada N., Tajiri K., Tsuji K., Ishikawa T., Ochi H. (2019). Real-life Practice Experts for HCC (RELPEC) Study Group and the HCC 48 Group (hepatocellular carcinoma experts from 48 clinics in Japan). Therapeutic potential of lenvatinib for unresectable hepatocellular carcinoma in clinical practice: Multicenter analysis. Hepatol. Res..

[19] Obi S., Sato T., Sato S., Kanda M., Tokudome Y., Kojima Y., Suzuki Y., Hosoda K., Kawai T., Kondo Y. (2019). The efficacy and safety of lenvatinib for advanced hepatocellular carcinoma in a real-world setting. Hepatol. Int..

[20] Rimini M., Shimose S., Lonardi S., Tada T., Masi G., Iwamoto H., Lai E., Burgio V., Hiraoka A., Ishikawa T. (2021). Lenvatinib versus sorafenib as first-line treatment in hepatocellular carcinoma: A multi-institutional matched case-control study. Hepatol. Res..

[21] Abd El Aziz M.A., Facciorusso A., Nayfeh T., Saadi S., Elnaggar M., Cotsoglou C., Sacco R. (2020). Immune Checkpoint Inhibitors for Unresectable Hepatocellular Carcinoma. Vaccines.

[22] Pfister D., Núñez N.G., Pinyol R., Govaere O., Pinter M., Szydlowska M., Gupta R., Qiu M., Deczkowska A., Weiner A. (2021). NASH limits anti-tumour surveillance in immunotherapy-treated HCC. Nature.

[23] European Association for the Study of the Liver (2018). EASL Clinical Practice Guidelines: Management of hepatocellular carcinoma. J. Hepatol..

[24] Llovet J.M., Lencioni R. (2020). mRECIST for HCC: Performance and novel refinements. J. Hepatol..

[25] Mathurin P., Bataller R. (2015). Trends in the management and burden of alcoholic liver disease. J. Hepatol..

[26] Fasanelli F., Giraudo M.T., Ricceri F., Valeri L., Zugna D. (2019). Marginal time-dependent causal effects in mediation analysis with survival data. Am. J. Epidemiol..

[27] Facciorusso A., Abd El Aziz M.A., Sacco R. (2019). Efficacy of Regorafenib in Hepatocellular Carcinoma Patients: A Systematic Review and Meta-Analysis. Cancers.

[28] Garuti F., Neri A., Avanzato F., Gramenzi A., Rampoldi D., Rucci P., Farinati F., Giannini E.G., Piscaglia F., Rapaccini G.L. (2021). The changing scenario of hepatocellular carcinoma in Italy: An update. Liver. Int..

[29] Kim S., Kim K.H., Kim B.K., Park J.Y., Ahn S.H., Kim D.Y., Kim S.U. (2021). Lenvatinib is independently associated with the reduced risk of progressive disease when compared to sorafenib in patients with advanced hepatocellular carcinoma. J. Gastroenterol. Hepatol..

[30] Nakano M., Kuromatsu R., Niizeki T., Okamura S., Iwamoto H., Shimose S., Shirono T., Noda Y., Kamachi N., Koga H. (2020). Primary Treatment with Molecular-Targeted Agents for Hepatocellular Carcinoma: A Propensity Score-matching Analysis. Hepatol. Commun..

[31] Tomonari T., Sato Y., Tanaka H., Mitsuhashi T., Hirao A., Tanaka T., Taniguchi T., Okamoto K., Sogabe M., Miyamoto H. (2021). Therapeutic efficacy of lenvatinib in nonviral unresectable hepatocellular carcinoma. JGH Open.

[32] Hatanaka T., Kakizaki S., Nagashima T., Namikawa M., Ueno T., Tojima H., Takizawa D., Naganuma A., Arai H., Harimoto N. (2021). Lenvatinib for hepatocellular carcinoma patients with nonviral infection who were unlikely to respond to immunotherapy: A retrospective, comparative study. Oncology.

[33] Mishra G., Majeed A., Dev A., Eslick G.D., Pinato D.J., Izumoto H., Hiraoka A., Huo T.-I., Liu P.-H., Johnson P.J. (2022). Clinical Utility of Albumin Bilirubin Grade as a Prognostic Marker in Patients with Hepatocellular Carcinoma Undergoing Transarterial Chemoembolization: A Systematic Review and Meta-analysis. J. Gastrointest. Cancer.

[34] Facciorusso A., Del Prete V., Crucinio N., Serviddio G., Vendemiale G., Muscatiello N. (2016). Lymphocyte-to-monocyte ratio predicts survival after radiofrequency ablation for colorectal liver metastases. World J. Gastroenterol..

[35] Llamosas-Falcon L., Shield K.D., Gelovany M., Manthey J., Rehm J. (2020). Alcohol use disorders and the risk of progression of liver disease in people with hepatitis C virus infection-a systematic review. Subst. Abus. Treat Prev. Policy.

[36] Luna-Cuadros M.A., Chen H.W., Hanif H., Ali M.J., Khan M.M., Lau D.T. (2022). Risk of hepatocellular carcinoma after hepatitis C virus cure. World J. Gastroenterol..

[37] Kai K., Koga H., Aishima S., Kawaguchi A., Yamaji K., Ide T., Ueda J., Noshiro H. (2017). Impact of smoking habit on surgical outcomes in non-B non-C patients with curative resection for hepatocellular carcinoma. World J. Gastroenterol..

[38] Costentin C.E., Mourad A., Lahmek P., Causse X., Pariente A., Hagège H., Dobrin A.S., Becker C., Marks B., Bader R. (2018). Hepatocellular carcinoma is diagnosed at a later stage in alcoholic patients: Results of a prospective, nationwide study. Cancer.

[39] Zamor P.J., deLemos A.S., Russo M.W. (2017). Viral hepatitis and hepatocellular carcinoma: Etiology and management. J. Gastrointest Oncol..

